# Myrrh Essential Oil Mitigates Renal Ischemia/Reperfusion-Induced Injury

**DOI:** 10.3390/cimb45020078

**Published:** 2023-02-01

**Authors:** Nancy S. Younis

**Affiliations:** 1 College of Clinical Pharmacy, King Faisal University, Al-Ahsa 31982, Saudi Arabia; nyounis@ku.edu.sa; 2 Department of Pharmacology, Zagazig University Hospitals, University of Zagazig, Zagazig 44519, Egypt

**Keywords:** anti-apoptotic, anti-inflammatory, antioxidant, essential oil, myrrh, renal ischemia/reperfusion

## Abstract

Background: Ischemia/reperfusion (I/R)-induced renal injury is a common reason for kidney injury in clinical settings; therefore, continuous investigation of novel nephroprotective agents is crucial. Myrrh, the oleoresin exudates generated by the genus *Commiphora*, display numerous pharmacological actions. This study tried to assess the preventive effects of myrrh essential oil against I/R-induced renal damage. Methods: Rats were randomized into five groups. In the sham group, the animals were subjected to bilateral renal artery separation with no occlusion. In the sham + myrrh group; the rats were administered myrrh essential oil and then treated similarly to the sham group. Renal I/R group: the animals were challenged with renal I/R. In the myrrh + renal I/R groups, rats were administered 50 or 100 mg/kg of myrrh essential oil orally for three weeks before being confronted with I/R. Results: Serum levels of renal function tests and renal injury biomarkers, including NGAL, KIM-1, and CysC, were amplified in the renal I/R group. Animals that experienced renal I/R exhibited elevated lipid peroxidation (MDA); declined SOD, CAT, and GPx activity; declined GSH content; augmented TLR4/NFκB gene expression; and subsequent enhancement of inflammatory mediators (TNF-α, IFN-γ, IL-1β, and IL-6). Myrrh reduced renal function tests and injury biomarkers and amended renal histological alterations. Pretreatment with myrrh reduced MDA, elevated the antioxidant enzymes’ activities and GSH content, and reduced the TLR4 and NFκB gene expression, leading to subsequent inflammation and apoptosis alleviation. Conclusions: The outcomes of the present investigation established the protective effect of myrrh essential oil against renal I/R via pointing out the antioxidant, anti-inflammatory, and anti-apoptotic effects of myrrh.

## 1. Introduction

Ischemia/reperfusion (I/R)-induced renal injury is a common reason for acute kidney injury that frequently occurs in clinical settings [1]. Treatment of acute kidney injury is mainly supportive treatment that affects the survivors’ quality of life, who may endure long-term outcomes, such as chronic kidney disease [2]. Ischemia, caused by clamping the renal vessels, reduces the supply of oxygen and nutrients to the renal tissue, whereas reperfusion worsens the state of oxidation and inflammation, resulting in necrosis and/or apoptosis [3]. The pathogenesis of I/R-induced renal damage is exceptionally complex, comprising intricate and interrelated events, including renal tubular damage, inflammation, oxidative stress, and vascular dysfunction, which ultimately lead to renal cell death [4]. Oxidative stress, directly and indirectly, manipulates several pathways leading to apoptosis, necrosis, fibrosis, and tissue damage progression, and eventually may result in renal dysfunction [5]. Additionally, excessive production of reactive oxygen species (ROS) induces an inflammatory response, which in turn causes leukocyte adhesion and migration to the ischemic area following the reperfusion [6,7]. Therefore, continuous exploration of novel possible nephroprotective agents, especially plants or naturally occurring compounds, is indispensable.

Natural constituents derived from plants have played a substantial role in drug discovery. Numerous plants’ secondary metabolites were explored to recognize novel compounds which showed several pharmacological actions, such as antifungal, antibacterial [8], and anticancer activities, among others [9]. *Commiphora molmol* (family Burseraceae) is a small thorny tree commonly distributed in Africa and Asia. Myrrh is the oleoresin exudate generated by the genus *Commiphora* [10]. One of the main components of Myrrh is the essential oil, which comprises 2 to 8% of the gum resin’s constituents. The essential oil comprises many compounds, especially of the sesquiterpenes type, particularly β-elemene, δ-elemene, β-bourbonene, α-bergamotene, germacrene A and B, furanoeudesma-1,3-diene, lindestren, and curzerene [11,12].

Myrrh’ oleoresin has been extensively used in wound-healing commercial products [10,13]. It showed numerous pharmacological actions, including analgesic [14], anti-inflammatory [14,15], anti-hyperlipidemic [14,16], antioxidant [15,17], anti-hyperglycemic [17], hepatoprotective [18], anti-osteoporotic [13], anti-ulcer [19], antiparasitic [20] antiviral [21], and anti-cancer [22] activity. Furthermore, myrrh alleviated ethanol-induced oxidative alterations of gastric ulceration [23], scopolamine-induced memory impairments [24], monosodium iodoacetate-induced osteoarthritis [25], and silicate-induced immune-mediated glomerulonephritis [26]. As for the protective action of myrrh against ischemia, an earlier study demonstrated the protective effects of myrrh essential oil on isoproterenol-induced myocardial infarction in rats [27]. Regarding renal actions, pre-treatment with *C. molmol* resin extract prevented methotrexate-induced kidney injury and attenuated oxidative stress and inflammation [28].

To the best of our knowledge, this is the first study exploring the protective activity of myrrh essential oil against renal I/R-induced injury. Therefore, in the current study, we tried to assess the protective effects of myrrh essential oil against I/R-induced renal damage with respect to its influence on the expression of the inflammatory and apoptotic markers and antioxidant activities. Moreover, this study investigated in more depth the mechanism of action of myrrh essential oil, proposing TLR4/ NF-κB as a pathway that might elucidate myrrh’s beneficial reno-protective effect.

## 2. Materials and Methods

### 2.1. Plant Acquisition and Myrrh Essential Oil Isolation and Analysis

As reported before by our laboratory team [27], the dried oleoresin of myrrh (*Commiphora molmol* Engler Family Burseraceae) was collected from local Markets and identified by taxonomists at King Saud University, Saudi Arabia. The dried oleoresin of *C. molmol* (300 g) was pulverized into coarse powder and then subjected to hydro-distillation to obtain the volatile fraction, which was then dried to attain the essential oil. The isolated myrrh essential oil was subjected to GC-MS analysis to identify major compounds, and the results were published previously [27].

### 2.2. Animals’ Acquisition and Ethical Approval

Wistar male rats (weight: 200–240 g) were procured from Experimental Animal Research Centre, King Saud University, Riyadh, KSA. Animals were maintained on a standard laboratory food and water ad libitum. Animals were retained in an adequately ventilated cage system (12 h light/dark cycle, 20.3–23.1 °C) during the investigation. Animal handling experiments and tests are harmonized with the procedures and regulations of the Ethical Conduct for the Use of Animals in Research at King Faisal University. Additionally, the Institutional Animal Care and Use Committee of King Faisal University permitted the experimental protocol (KFU-REC-2021—DEC-EA000235).

### 2.3. Experimental Design

Rats were randomized into five groups after one week of acclimatization (*n =* 6). The first group was the sham group, in which the animals were subjected to bilateral renal artery separation with no occlusion. The second group was the sham + myrrh group, in which rats were administered myrrh essential oil (100 mg/kg) dissolved in 1% carboxymethylcellulose in saline orally using a gastric gavage for three weeks. Then, animals were treated similarly to the sham group. In the renal I/R group, the animal was challenged with renal ischemia/reperfusion (I/R) surgery. In myrrh + renal I/R groups, the rats were administered myrrh essential oil at 50 mg/kg or 100 mg/kg [27] orally for three weeks before being confronted with renal ischemia/reperfusion (I/R) surgery.

### 2.4. Renal Ischemia/Reperfusion (I/R) Surgery Procedures

Rats were fasted for eight hours, then anesthetized using isoflurane with oxygen (2%, 0.5 L/h) and positioned on a surgical heating pad. Under sterilized circumstances, a midline cut was executed to separate the kidneys located within the retroperitoneal area. The left and right pedicles were pinched for 30 min using non-traumatic vascular clamps to establish renal ischemia [29]. The pale kidney color designated renal ischemia. After 30 min, the clamps were detached, and the kidneys were observed for a few minutes until the color turned reddish-brown, indicating reperfusion manifestation, and both incisions were sutured. Sham groups did not undergo renal pedicle occlusion. Forty-eight hours after renal I/R surgery, animals were anesthetized, and blood samples were attained via cardiac punctures and centrifuged (20 min, 5000× *g*) to obtain serum. Serum samples were stored at −20 °C to be used later for different biochemical inspections. One kidney was rapidly dissected, homogenized, and kept at −20 °C for biochemical studies, and the other was conserved in 10% formalin for histopathological and immunohistochemical investigation.

### 2.5. Histopathological and Immunohistochemistry Investigations

For histopathological examination, tissue samples were fixed in formalin for 24 h; paraffin beeswax tissue blocks were cut to acquire 4 μm thick sections. Renal sections were collected on glass slides, deparaffinized, and stained using Hematoxylin and Eosin (H&E) to be inspected under a light microscope. Two pathologists (blinded to the experiment) evaluated different kidney sections with a specific scale to assess the renal tubulointerstitial damage grade using at least ten fields for each renal section. The scoring scale designated 0 (no damage), 1 (less than 10%), 2 (11–25%), 3 (26–45%), 4 (46–75%), and 5 (more than 76%) as indicated before by Tan et al. [30].

As for the immunohistochemistry technique, the expression of TLR4 and NFκB was inspected as decribed previously [31]. Renal sections were blocked using 3% hydrogen peroxide in methanol and washed with PBS three times. Then, the sections were incubated with TLR4 and NFκB antibodies (1:100, Thermo Fisher Scientific, Cambridge, UK) overnight at 4 °C, followed by the addition of the goat anti-rabbit-horseradish peroxidase (HRP)-conjugated IgG antibody (1:1000; Cat. no. ab6721; Abcam, Eugene, OR, USA) for 1 h at 37 °C. Finally, the tissue sections were developed with 1% diaminobenzidine, counterstained with 1% hematoxylin, and mounted with neutral gum. A microscope equipped with a digital camera was utilized to capture the renal sections. NIS-Elements software was used for quantitative analysis. The area of the immunohistochemistry reaction was selected, and then the average optical density in the designated area of each photograph was assessed. Positive cells were counted under 400× magnification, observing ten consecutive non-overlapping fields per animal in a blinded manner.

### 2.6. Assessment of the Kidney Function

Kidney function was assessed by measuring serum levels of creatinine (Cr, Cat. No. ab700460), uric acid (Cat. No. ab65344), blood urea nitrogen (BUN, Cat. No. ab83362), and lactate dehydrogenase (LDH, Cat. No. ab102526), using the specified colorimetric or ELISA kit acquired from Abcam Inc. (Waltham, MA, USA) and implemented following the manufacturer’s instructions.

### 2.7. Assessment of the Kidney Injury Biomarkers

The serum levels of cystatin C (CysC, Cat. No. ab201281), neutrophil gelatinase-associated lipocalin (NGAL, Cat. No. ab119597), and kidney injury molecule-1 (Kim-1, Cat. No. ab119597) were measured using the specified ELISA kits and analyzed by spectrophotometry at 450 nm.

### 2.8. Assessment of the Kidney Oxidative Stress

Malondialdehyde (MDA, Cat. No. ab238537), glutathione peroxidase (GPx, Cat. No. ab102530), and glutathione content (GSH, Cat. No. ab239727) were assessed in renal tissue homogenate using ELISA kits, which were obtained from Abcam Inc. (Waltham, MA, USA). In addition, superoxide dismutase (SOD, Cat. No. MBS036924) and catalase (CAT, Cat. No. MBS726781) were evaluated in renal homogenate using ELISA kits that were purchased from MyBioSource (San Diego, CA, USA).

### 2.9. Gene Expression by Real-Time PCR (qPCR)

Gene expression of TLR4 and NFκB and the apoptotic markers, Bax and Bcl2, was quantified via real-time PCR (qPCR), while consuming the primers’ sequences in compliance with the method described elsewhere [32]. Concisely, RNA was isolated and purified using the Trizol reagent kit and then reverse transcribed using the reverse transcription-polymerase chain reaction (RT-PCR) kit (TaKaRa, Kusatsu, Shiga, Japan), agreeing with the manufacturer’s procedures. qPCR was applied using SYBR ExScript RT-PCR kit, and quantification examinations were completed via an Opticon-2 Real-time PCR reactor (MJ Research, Capital Court, Reno, NV, USA). qPCR results were obtained using Step PE Applied Biosystems (Waltham, MA, USA) software. Target gene expression levels were evaluated and correlated with that of β-actin as the reference gene, and the results are shown in figures as relative expression levels. The primer sequences used in this study: TLR4 sense: 5′- CATGA CATCCCTTATTCAACCAAG-3′, antisense: 5′-GCCATGCCTTGTCTTCAATTG-3′; NFκB sense: 5′-ATCATCAACATGAGAAACGATCTGTA-3′, antisense: 5′-CAGCGGTCCAGA AGACTCAG-3′; Bax sense: 5′-GTGGTTGCCCTCTTCTACTTTG-3′, antisense: 5′-CAAAA GATGGTCACTGTCTGC-3′; Bcl-2 sense: 5′-CCGGGAGATCGTGATGAAGT-3′, antisense: 5′-ATCCCAGCCTCCGTTATCCT-3′; β-Actin sense: 5′-TGCTATGTTGCCCTAGACTTC G-3′, antisense: 5′-GTTGGCATAGAG GTCTTTACGG-3′. β-actin expression was used for sample normalization, whereas the 2^−ΔΔCT^ equation was used for relative expression determination.

### 2.10. Assessment of the Kidney Inflammatory Mediators

Inflammation markers, including interleukin 1 beta (IL-1β, Cat. No. ab100768), interleukin 6 (IL-6, Cat. No. ab100772), tumor necrosis factor-alpha (TNF-α, Cat. No. ab239425), interferon-gamma (IFN-γ, Cat. No. ab46107) and interleukin 10 (IL-10, Cat. No. ab214566) were measured in renal tissue homogenate using ELISA Kits which were attained from Abcam Inc. (Waltham, MA, USA) according to the manufacturer protocols.

### 2.11. Statistical Analysis

Data were presented as mean ± SD. For multiple comparisons, one-way ANOVA followed by Tukey–Kramer as a post hoc test was performed. The 0.05 level of probability was used as the significance level. All statistical analyses were performed using Graph Pad software (version 8, San Diego, CA, USA).

## 3. Results

### 3.1. Myrrh Essential Oil Constituents

As shown in a previous study we published [27], 104 components were separated from the essential oil, from which 44 components were identified. These identified compounds corresponded to 94.156% of the total separated components obtained from Myrrh essential oil. Sesquiterpenes represent the main portion of myrrh essential oil’s constituents with 38 compounds, representing 89% of the total area percentage, including copaene, β-elemene, β-caryophyllene, curzerene, α-cadinene, and furanoeudesma 1,3-diene. Several oxygenated compounds, furanosesquiterpenens (32.355%), and many compounds with different cyclization patterns were also recognized.

### 3.2. Myrrh Essential Oil Improved Renal Function Subsequent to Renal I/R

**Figure 1:**
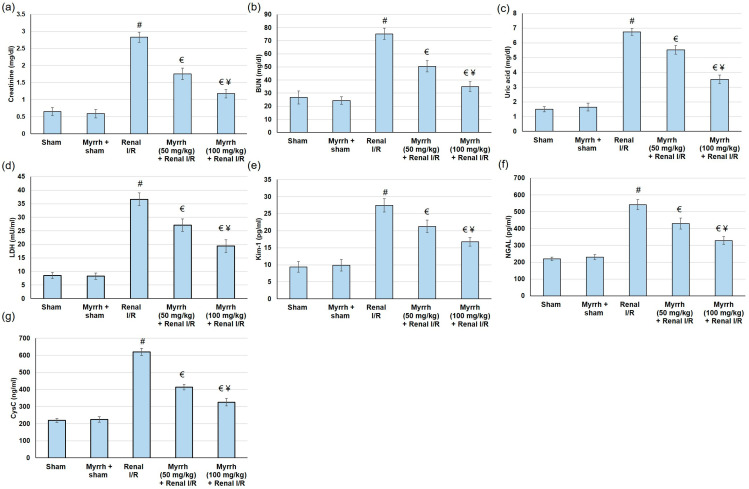
The assessment of myrrh essential oil (50 and 100 mg/kg) administration for three weeks prior to renal ischemia/perfusion (I/R) through the renal function tests: (**a**) creatinine, (**b**) BUN, (**c**) uric acid, (**d**) LDL; renal injury biomarkers: (**e**) Kim-1, (**f**) NGAL, and (**g**) CysC. All values are expressed as mean ± SD. # indicates a statistically significant difference from the sham group, € indicates a statistically significant difference from the renal I/R group, and ¥ indicates a statistically significant difference from myrrh 50 mg/kg + renal I/R group (*p* < 0.05) using one-way ANOVA followed by Turkey’s post hoc test (*n* = 6).

Assessment of the renal function following renal I/R was performed by measuring several renal biomarkers. Blood urea nitrogen (BUN) and creatinine (Cr) were maintained in the blood, indicating less renal filtration. Similarly, LDH was released into the bloodstream subsequent to I/R-induced renal injury. As shown in Figure 1, serum levels of Cr, BUN, uric acid, and LDH were considerably amplified in the renal I/R group compared to the sham group. On the other hand, myrrh essential oil (50 and 100 mg/kg) administration prior to renal I/R significantly depressed the augmented levels of Cr, BUN, uric acid, and LDH in relation to the I/R group (*p* < 0.05). Myrrh essential oil (50 and 100 mg/kg) caused percentage reductions of 38.0% and 58.5% for Cr, 32.86% and 53.5% for BUN, 17.95% and 47.65% for uric acid, and 26.06% and 47.16% for LDH (Figure 1a–d). These outcomes specify that myrrh administration prior to renal I/R may limit renal-function deterioration associated with I/R.

### 3.3. Myrrh Essential Oil Deterred Renal Injury Biomarkers

Several renal injury biomarkers, such as neutrophil gelatinase-associated lipocalin (NGAL), kidney injury molecule-1 (KIM-1), vanin-1, liver-type fatty acid-binding protein (L-FABP), and cystatin C (CysC) are regularly used to indicate renal proximal tubule injury [33,34]. In the current study, the serum levels of NGAL, Kim-1, and CysC were significantly augmented in the renal I/R group compared to the sham, as represented in Figure 1e–g. On the other hand, myrrh essential oil (50 and 100 mg/kg) administration considerably reduced the amplified levels of NGAL, Kim-1, and CysC, causing percentage reductions of 20.74% and 39.33% for NGAL, 22.44% and 38.83% for Kim-1, and 33.03% and 47.40% for CysC. These outcomes specify that myrrh administration prior to renal I/R may limit renal injury, as indicated by renal injury markers.

### 3.4. Myrrh Essential Oil Amended Renal Histological Alterations Subsequent to Renal I/R

**Figure 2:**
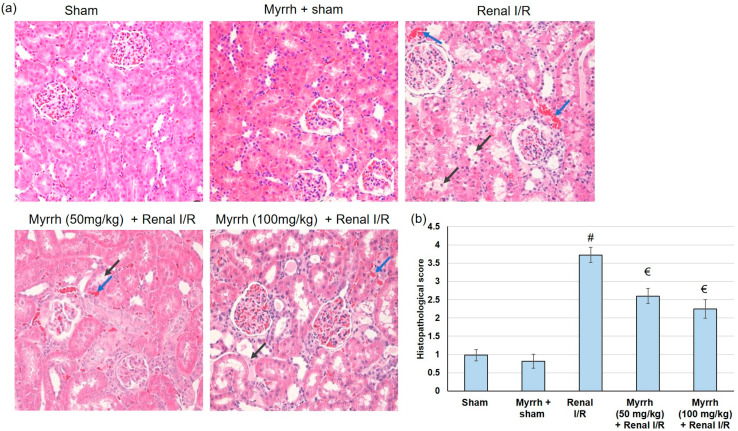
The assessment of myrrh essential oil (50 and 100 mg/kg) administration for three weeks prior to renal ischemia/perfusion (I/R) in histopathological analysis: (**a**) renal sections stained with hematoxylin and eosin (H&E) and (**b**) quantitative assessment of renal damage score. Blue arrows show renal proteinaceous casts and edema, and black arrows show masses of necrotic cellular debris. # indicates a statistically significant difference from the sham group, € indicates a statistically significant difference from the renal I/R group.

As shown in Figure 2, renal histological changes were assessed by H&E staining. No observable kidney pathological injury was witnessed in the renal tissues obtained from the sham or myrrh + sham groups, whereas the I/R group displayed the representative features of renal injury. Renal interstitial congestion proteinaceous casts, edema (blue arrow), and large masses of necrotic cellular debris (black arrows) were spotted. On the other hand, administration of myrrh prior to renal I/R considerably mitigated the degree of renal damage and fundamentally preserved the kidney integrity, whereas no difference between the two doses of myrrh was observed.

### 3.5. Myrrh Essential Oil Deterred Renal Oxidative Stress Subsequent to Renal I/R

**Figure 3:**
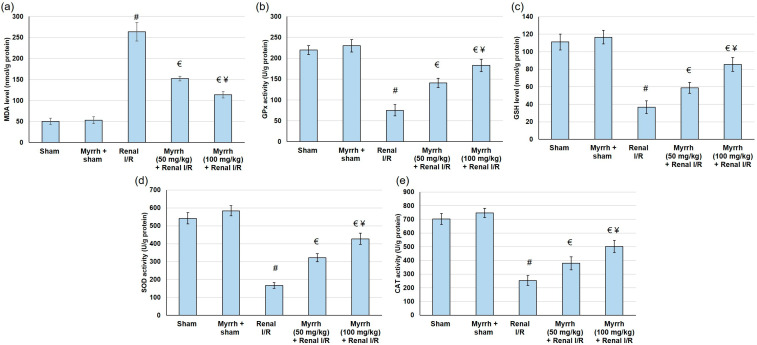
An assessment of myrrh essential oil (50 and 100 mg/kg) administration for three weeks prior to renal ischemia/perfusion (I/R). Renal oxidative stress markers: (**a**) MDA, (**b**) GSH, (**c**) GPx, (**d**) SOD, and (**e**) CAT. All values are expressed as mean ± SD. # indicates a statistically significant difference from the sham group, € indicates a statistically significant difference from the renal I/R group, and ¥ indicates a statistically significant difference from myrrh 50 mg/kg + renal I/R group (*p* < 0.05) using one-way ANOVA followed by Turkey’s post hoc test (*n* = 6).

MDA is one of the final products of polyunsaturated fatty acids’ peroxidation in the cells and is thus commonly used as a lipid peroxidation indicator [35]. In the current study, the MDA level exhibited a significant elevation in animals that exhibited renal I/R compared to sham groups. In comparison, pretreatment with myrrh essential oil (50 and 100 mg/kg) caused a reduction in the MDA level (42.14% and 56.87%, respectively) compared to the renal I/R group. One of the main contributors to I/R-induced renal damage is oxidative stress [36]. In the current study, rats that experienced I/R demonstrated markedly declined SOD, CAT, and GPx enzyme activities and GSH content in renal tissue. Myrrh (50 and 100 mg/kg) enhanced the activities SOD, CAT, and GPx, and increased GSH content in renal tissue (*p* < 0.05), as displayed in Figure 3. All these results indicate that myrrh deterred renal oxidative stress subsequent to renal I/R via augmenting antioxidant enzymes’ activities and GSH content, and lowering the MDA level.

### 3.6. Myrrh Essential Oil Deterred TLR4/NFκB Pathway Activation Subsequent to Renal I/R

**Figure 4:**
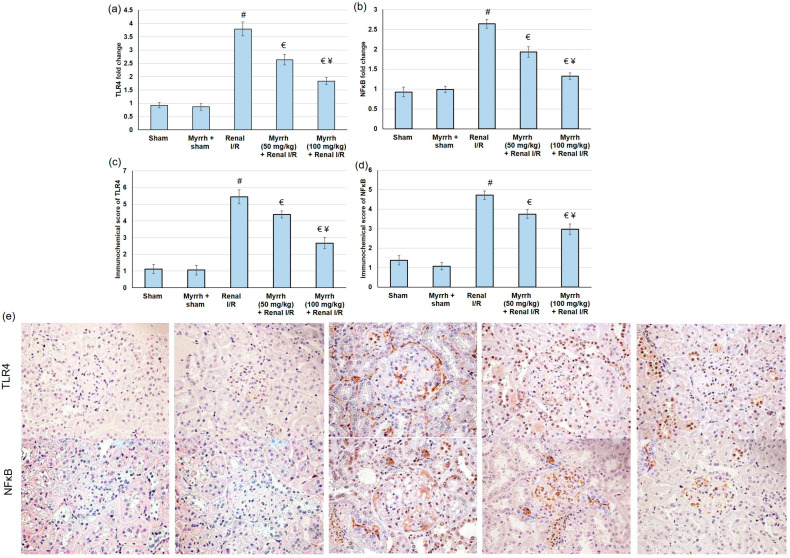
The assessment of myrrh essential oil (50 and 100 mg/kg) administration for three weeks prior to renal ischemia/perfusion (I/R) via gene expression analysis of (**a**) TLR4 and (**b**) NFκB; renal immunohistochemical scoring assays: (**c**) TLR4 and (**d**) NFκB; and immunohistochemical images for (**e**) TLR4 and NFκB. All values are expressed as mean ± SD. # indicates a statistically significant difference from the sham group, € indicates statistically substantial from the renal I/R group, and ¥ indicates a statistically significant difference from myrrh 50 mg/kg + renal I/R group (*p* < 0.05) using one-way ANOVA followed by Turkey’s post hoc test (*n* = 6).

Renal injury triggered by I/R produces numerous damage-associated molecular patterns (DAMPs) proteins, which signal through TLRs, mainly TLR4, to activate multiple inflammatory mediators [37]. NFκB is an essential downstream effector of TLR4 signaling. Therefore, in the current study, we evaluated both TLR4 and NFκB by real-time PCR and immunohistochemistry. Compared to the sham group, TLR4 and NFκB gene expression was significantly augmented in the renal I/R group. TLR4 and NFκB gene expression declined considerably in rats pretreated with myrrh essential oil compared to renal I/R animals, as shown in Figure 4.

### 3.7. Myrrh Essential Oil Deterred Renal Inflammation Subsequent to Renal I/R

**Figure 5:**
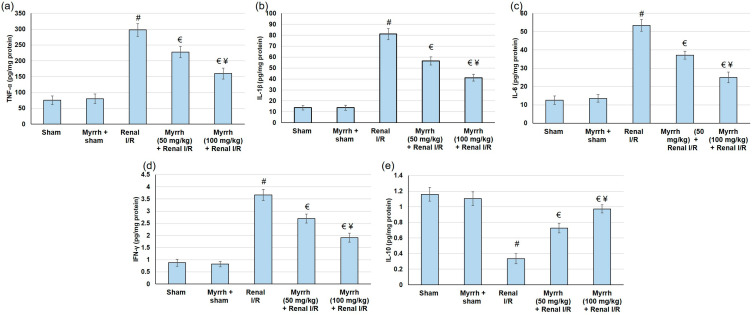
The assessment of myrrh essential oil (50 and 100 mg/kg) administration for three weeks prior to renal ischemia/perfusion (I/R) regarding inflammatory mediators: (**a**) TNF-α, (**b**) IL-1β, (**c**) IL-6, (**d**) IFN-γ, and (**e**) IL-10. All values are expressed as mean ± SD. # indicates a statistically significant difference from the sham group, € indicates statistically substantial from the renal I/R group, and ¥ indicates a statistically significant difference from myrrh 50 mg/kg + renal I/R group (*p* < 0.05) using one-way ANOVA followed by Turkey’s post hoc test (*n* = 6).

During I/R, TLR4/ NFκB activation enhances proinflammatory responses, shown by elevations in several inflammatory indicators. The current investigation examined the inflammation status via recognizing several cytokines and inflammatory mediators. TNF-α, IFN-γ, IL-1β, and IL-6 renal levels were significantly amplified (*p* < 0.05) in animals that experienced I/R. In contrast, myrrh administration prior to renal I/R clearly deterred TNF-α, IFN-γ, IL-1β, and augmented IL-6 renal levels (Figure 5). Alternatively, IL-10, a cytokine which exerts protective actions against inflammatory injury, declined (*p* < 0.05) subsequent to I/R surgery, whereas myrrh noticeably increased the IL-10 level.

### 3.8. Myrrh Essential Oil Deterred Renal Apoptosis Subsequent to Renal I/R

**Figure 6:**
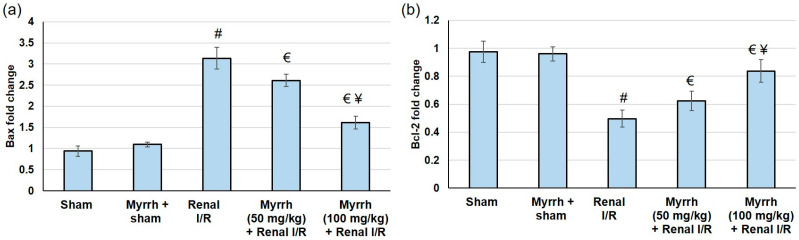
The assessment of myrrh essential oil (50 and 100 mg/kg) administration for three weeks prior to renal ischemia/perfusion (I/R) regarding the apoptosis mediators (**a**) Bax and (**b**) Bcl2 gene expression. All values are expressed as mean ± SD. # indicates a statistically significant difference from the sham group, € indicates statistically substantial from the renal I/R group, and ¥ indicates a statistically significant difference from myrrh 50 mg/kg + renal I/R group (*p* < 0.05) using one-way ANOVA followed by Turkey’s post hoc test (*n* = 6).

Animals subjected to renal I/R surgery presented exaggerated apoptosis, comprising enhanced Bax gene expression and reduced Bcl2 gene expression; see Figure 6. Pretreatment with myrrh resulted in apoptosis alleviation, as established by lowered Bax gene expression and increased Bcl2 gene expression, confirming the anti-apoptotic effect of the compound.

## 4. Discussion

Myrrh has been traditionally used to treat rheumatoid arthritis, sinusitis and cough, gum and gingival problems, and sore throat [38]. It is a natural remedy for various conditions. However, the renal protective effect of myrrh has not been explored before. Ischemia-reperfusion (I/R) injury is the leading cause of acute kidney injury, during which a rapid deterioration in renal function occurs, causing high morbidity and mortality among patients [39,40]. In the current study, serum levels of Cr, BUN, uric acid, and LDH were amplified in the renal I/R group compared to the sham group. Myrrh improved renal function though, as displayed by the depressed serum levels of Cr, BUN, uric acid, and LDH. Furthermore, myrrh essential oil deterred renal injury biomarkers by decreasing serum levels of NGAL, KIM-1, and CysC; and amended renal histological alterations associated with renal I/R. NGAL is an early biomarker of AKI that is manufactured in the distal nephron, and its synthesis is elevated as a response to renal injury [41,42]. Furthermore, recent studies revealed that an elevated NGAL level is a risk factor for oxidative stress in hemodialysis patients [43]. These outcomes designate that myrrh essential oil administration prior to renal I/R may limit renal deterioration caused by I/R. Similarly, an earlier study showed that myrrh provided protection against methotrexate-induced acute kidney injury, as evidenced by decreasing renal function tests [28].

The underlying pathophysiological mechanisms involved in renal I/R include the release of reactive oxygen species (ROS), generation of pro-inflammatory mediators, calcium overload, activation of apoptotic genes [3], and vascular dysfunction [44]. ROS has a detrimental effect on the kidney tissues, thereby playing a decisive role in the renal injury pathophysiological processes. Oxygen radicals triggered by I/R cause lipid peroxidation and destroy cell and organelle membranes, thereby disturbing the renal tissue structure and function [45]. The results of the current study displayed an elevation in the lipid peroxidation (MDA) level, accompanied by lowered SOD, CAT, and GPx enzyme activity, and GSH content, in renal tissue in animals that experienced renal I/R. However, pretreatment with myrrh essential oil (50 and 100 mg/kg) caused a reduction in lipid peroxidation (MDA), together with increases in SOD, CAT, and GPx enzymes’ activities and an increase in GSH content in renal tissue. All these results together indicate that myrrh deterred renal oxidative stress subsequent to renal I/R via augmenting antioxidant enzymes’ activities, GSH content, and lowering the level of MDA—in harmony with earlier reports which showed that *Commiphora molmol* attenuated oxidative stress in diverse diseases such as tilmicosin-induced cardiotoxicity [46], ethanol-induced gastric ulceration [23], acetic acid-induced ulcerative colitis [15], hyperammonemia [47], and diethylnitrosamine/phenobarbital-induced hepatocarcinogenesis [48], among other animal disease models.

In terms of inflammation, ischemia-induced hypoxia in the renal tissue leads to the vigorous local production of inflammatory cytokines [49]. These cytokines can initiate defensive physiological activities to isolate and inhibit tissue damage and/or further aggravate organ damage and dysfunction by inducing free radicals to produce and recruit inflammatory cells [26]. I/R generates numerous damage-associated molecular patterns (DAMPs) proteins, which signal through TLRs, mainly TLR4, to activate numerous inflammatory mediators [37]. NFκB is one of the essential downstream effectors of the TLR4 signaling [50]. TLR4/NFκB pathway activation enhances pro-inflammatory responses, thereby elevating several inflammatory markers [51,52]. At the same time, IL-10 suppresses the production of pro-inflammatory cytokines, renal dysfunction, and the expression of pro-apoptosis factors after I/R [53]. In the current study, TLR4 and NFκB gene expression were significantly augmented in the renal I/R group. As a result of TLR4 and NFκB activation, renal levels of TNF-α, IFN-γ, IL-1β, and IL-6 were greatly amplified in animals that experienced I/R. Numerous earlier studies exposed the activation of the TLR4/NFκB pathway as an essential pathway responsible for the damage happening during I/R, and that suppressing the TLR4/NFκB pathway revealed renal protection from injury caused by I/R [54,55,56].

On the other hand, myrrh administration prior to renal I/R evidently deterred TLR4 and NFκB gene expression, leading to subsequent drops in TNF-α, IFN-γ, IL-1β, and IL-6 renal levels. Moreover, myrrh noticeably augmented the IL-10 level, indicating that the essential oil deterred the TLR4/NFκB pathway, resulting in the mitigation of renal inflammation. These outcomes are in agreement with earlier studies that verified the anti-inflammatory effects of myrrh against formalin-induced paw edema [14], ovariectomy-induced bone loss [13], monosodium iodoacetate-induced osteoarthritis [25], wound healing [57], and silicate induced immune-mediated glomerulonephritis [26]. Additionally, animals experimented with renal I/R surgery presented exaggerated apoptosis, comprising enhanced Bax gene expression, and reduced Bcl2 gene expression. Pretreatment with myrrh essential oil resulted in apoptosis alleviation, as established by lowered Bax and increased Bcl2 gene expression levels, confirming the anti-apoptotic effect of the oil. Similarly, pretreatment with myrrh exhibited an anti-apoptotic effect via up-regulating Bcl-2 in the kidney of methotrexate-induced renal toxicity rats [38]. Several natural oils, such as lavender oil [58], and natural compounds, such as senkyunolide I [59], attenuated renal I/R injury via suppressing apoptosis.

## 5. Conclusions

The outcomes of the present investigation established the protective effect of myrrh essential oil against renal I/R. Myrrh essential oil administration prior to renal I/R improved renal function, as displayed by the depressed levels of Cr, BUN, uric acid, and LDH; and the limited renal deterioration. The existing investigation outcomes revealed that mitigating oxidative stress by up-regulating the antioxidant enzyme activity contributes to myrrh’s protective effect against renal I/R-induced oxidative stress. Additionally, pretreatment with myrrh essential oil inhibited the TLR4/NF-κB pathway, a mechanism that might be behind myrrh’s beneficial protective effect. Inhibiting the TLR4/NF-κB pathway was reflected by reductions in multiple inflammatory and apoptotic markers. Bringing together all these outcomes, the present study showed for the first time the renal-protective activity of myrrh essential oil against renal I/R-induced injury, pointing out the antioxidant, anti-inflammatory, and anti-apoptotic actions of myrrh essential oil.

## Data Availability

Not applicable.
